# Frequent callers to telephone helplines: new evidence and a new service model

**DOI:** 10.1186/s13033-016-0076-4

**Published:** 2016-05-31

**Authors:** Jane Pirkis, Aves Middleton, Bridget Bassilios, Meredith Harris, Matthew J. Spittal, Izabela Fedszyn, Patty Chondros, Jane Gunn

**Affiliations:** Centre for Mental Health, Melbourne School of Population and Global Health, The University of Melbourne, Melbourne, Australia; Department of General Practice, Melbourne Medical School, The University of Melbourne, Melbourne, Australia; School of Population Health, The University of Queensland, Brisbane, Australia; Policy and Epidemiology Group, Queensland Centre for Mental Health Research, Brisbane, Australia

**Keywords:** Telephone, Helpline, Crisis, Frequent callers

## Abstract

**Background:**

This paper describes a program of work designed to inform a service model to address a challenge for telephone helplines, namely frequent callers.

**Methods:**

We conducted a systematic literature review and four empirical studies that drew on different data sources—(a) routinely collected calls data from Lifeline, Australia’s largest telephone helpline; (b) data from surveys/interviews with Lifeline frequent callers; (c) data from the Diagnosis, Management and Outcomes of Depression in Primary Care *(diamond)* study; and (d) data from Australia’s National Survey of Mental Health and Wellbeing.

**Results:**

Frequent callers represent 3 % of callers but make 60 % of calls. They are isolated and have few social supports but are not “time wasters”; they have major mental and physical health problems and are often in crisis. They make use of other services for their mental health problems. The circumstances under which they use telephone helplines vary, but current service models reinforce their calling behaviour.

**Conclusions:**

The findings point to a service model that might better serve the needs of both frequent callers and other callers. The model involves offering frequent callers an integrated, tailored service in which they are allocated a dedicated and specially trained telephone crisis supporter (TCS), and given set calling times. It also involves promoting better linkages between telephone helplines and other services that provide mental health care, particularly general practitioners (GPs) and other primary care providers. The next step is to refine and test the model.

## Background

Around the world, telephone helplines exist to provide support to callers who are going through a crisis (defined as a transient state of psychological disequilibrium during which their usual coping mechanisms are disrupted) [1, 2]. These helplines typically target people who may be experiencing mental health problems (e.g., depression or anxiety), have suffered abuse or trauma, be facing immediate stressors, or be feeling socially isolated. Often they have a particular emphasis on providing help for people who are at immediate risk of suicide. Helplines aim to assist callers to develop strategies to deal with the circumstances underpinning the crisis, and, where appropriate, suggest services that may offer more specialised, professional support. Their workers (termed telephone crisis supporters, or TCSs, in this paper) are trained to develop a rapport with callers, responding to them in a way that is respectful and non-judgemental. Some helplines are directive, offering strategies and advice, and others are non-directive, providing someone who listens but does not suggest interventions. Usually, but not always, callers to helplines remain anonymous.

Assistance from telephone helplines is generally intended as a one-off or time-limited intervention [1]. Some people, however, make numerous calls to helplines, sometimes over a relatively short space of time and sometimes for prolonged periods. In this paper, we generally refer to these callers as “frequent callers” [3, 4], noting that they have also been described as “chronic callers” [2, 5–12] and “repeat callers” [13, 14]. Frequent callers present a challenge for telephone helplines which aim to achieve the best outcome for all callers but have to do so with finite resources. Responding to frequent calls from one caller may mean that several other callers cannot connect with the service. It may also encourage a dependency on the service in frequent callers that is not in their best interests [15]. Telephone helplines have been struggling with this issue for over 50 years and have not yet solved it [16].

In Australia, the largest telephone helpline is Lifeline. Lifeline is a not-for-profit organisation that provides access to crisis support, suicide prevention and mental health services. Key amongst these is its 13 11 14 telephone line, which offers 24-hour crisis support. It also offers an online crisis support chat service (Crisis Chat) within a more restricted range of hours for people who are feeling overwhelmed and having difficulty coping, but may not feel comfortable speaking to someone over the telephone. Both services are national, and both are staffed by highly-trained, dedicated TCSs [17].

Like other telephone helplines, Lifeline has grappled with the issue of frequent callers for some time, notionally identifying them as callers who call 20 times or more in a month. In 2014, Lifeline received 884,000 crisis calls and answered 735,000 (83 %) of them [17]. Acknowledging that this response rate would be higher if the demand from frequent callers could be reduced, the Lifeline Research Foundation commissioned us to conduct a program of work on frequent callers. Specifically, we were asked to describe the profile of frequent callers to Lifeline crisis support services, and develop a service model that responds to them. The program of work included a systematic literature review [18] and four empirical studies [19–22]. The findings from each of these have been or will be reported elsewhere, but this paper synthesises them, and uses these findings to inform a discussion about the principles and potential features of a service model that might free up resources for episodic and one-off callers while at the same time meeting the needs of frequent callers. The paper does this with a view to providing lessons for others who may also be seeking to address the issue of frequent callers.

## Methods

### Systematic literature review

We searched Medline and ProQuest for articles relating to frequent callers to telephone helplines that were published between 1960 and 2012. Full text articles which presented empirical data on callers who made multiple calls to helplines were included in the review [18].

### Empirical studies

The empirical studies drew on data from four different sources: (a) routinely collected Lifeline calls data [21]; (b) data from a purpose-designed survey/interview study with frequent callers to Lifeline [22]; (c) data from the Diagnosis, Management and Outcomes of Depression in Primary Care *(diamond)* study [20]; and (d) data from Australia’s National Survey of Mental Health and Wellbeing (NSMHWB) [19]. The first two data sources were Lifeline-specific; the latter two provided additional perspectives on patterns of usage of telephone helplines more generally. The methods used in these studies are described below.

#### Routinely collected Lifeline calls data

Lifeline provided us with data on calls made to its 13 11 14 number between December 2011 and May 2013 [21]. Certain data are routinely captured for each call including: telephone number; call date, time, region of origin, duration and type (crisis versus non-crisis); and caller demographics and presenting issues.

Before releasing the data to us, Lifeline encrypted the telephone numbers associated with each call. We assumed that calls from the same (encrypted) number were made by the same person, and aggregated call level data up to the person level. This allowed us to look across time at calling patterns for individuals, and to identify frequent callers. We took Lifeline’s rule of thumb of 20 calls per month as a guide, and scaled this up or down so that any individual who made 0.667 calls per day in any period longer than a week (4.7 calls in 7 days, 20 calls in 30 days, 40 calls in 60 days, and so on) was regarded as a frequent caller.

#### Data from a purpose-designed survey/interview study with frequent callers to Lifeline

We worked with two New South Wales Lifeline centres (Harbour to Hawkesbury and Northern Beaches) to investigate the factors that influence callers’ calling patterns, and the long term benefits of these calls [22]. Between February and July 2015, supervisors at these centres who had received specific training administered a brief survey to eligible crisis callers at the conclusion of their call. The survey included a question about callers’ calling behaviour in the past month. Those who indicated that they had contacted Lifeline only once were classified as one-off callers, those who said that they had done so between two and 19 times were regarded as episodic callers, and those who reported that they had done so 20 or more times were deemed to be frequent callers.

The survey ended with an invitation to participate in a more detailed interview. Those who accepted this were later asked a set of open-ended questions that explored their experiences with Lifeline, the reasons why they continued to call, and their use of other health services. The interviews were conducted by a member of our study team over the telephone, audio-recorded and transcribed. We analysed interview data using a thematic analysis methodology.

#### Data from the diamond study

The *diamond* study involves a sample of 789 patients with depressive symptoms who were recruited via 30 general practices in 2005 and have been interviewed annually since then [23]. We used data from the first year of follow-up, during which participants completed postal surveys at 3 month intervals (three, six, nine and 12 months) [20]. The surveys asked participants to provide information on their socio-demographic characteristics, mental and physical health, and use of health and other services. Among the latter group of questions was one that asked how often participants had used telephone helplines for depression, stress or worries in the past 3 months. Those who indicated that they had done so once a week or more were regarded as frequent callers.

#### Data from Australia’s NSMHWB

The NSMHWB was conducted in 2007 by the Australian Bureau of Statistics and collected data from a representative sample of 8841 adults [24]. Respondents were interviewed in their homes, and asked a range of questions about their socio-demographic details, their mental health status, their levels of suicidality, and their use of a range of services. All NSMHWB respondents were asked “Did you ever use a telephone counselling service (such as Lifeline) for problems with your mental health?” Those who answered “yes” were then asked “In the past 12 months, how many times did you use a telephone counselling service?” Using this question, we classified respondents into non-callers, one-off callers and repeat callers.

## Results

### Systematic literature review

We identified 21 articles that reported on 19 separate studies, 11 of which were call record audits [2, 6, 9, 12, 15, 16, 25–30], five of which were follow-up surveys of callers [1, 31–35], and three of which were intervention studies [5, 13, 36].

The published studies provided us with some insights into frequent callers. The studies suggested that frequent callers were more likely to be male and unmarried than other callers, but that, in the main, other key variables like age, mental health conditions or suicidality were unrelated to calling patterns. This led to suggestions about strategies that might meet the needs of frequent callers, including limiting the number and duration of calls permitted, assigning a specific TCS to the caller, implementing face-to-face contact, initiating contact with the caller rather than waiting for him or her to call, providing short-term anxiety and depression treatment programs over the telephone, and creating individualised management plans. The first two strategies were evaluated in intervention studies and found to show promise in terms of reducing the number and/or duration of calls made by frequent callers [5, 36] and, to a lesser extent, in terms of reducing callers’ suicidality [13]. The remaining strategies are as yet untested.

These findings should be interpreted in the context of the limitations of the reviewed studies, which were quite significant. For example, the studies used a variety of definitions of frequent callers, most commonly using the very inclusive definition of “more than one call”, and not uncommonly failing to use a definition at all. Their samples were often small and/or unrepresentative, and only two compared frequent callers with other callers. Most of the studies were relatively dated; over two-thirds were conducted before 2000. We concluded that more up-to-date, rigorous research is needed in this area.

### Empirical studies

#### Routinely collected Lifeline calls data

**Fig. 1:**
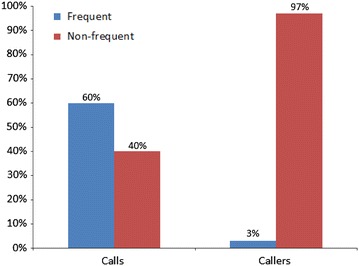
Distribution of Lifeline calls and callers, December 2011–May 2013

In total, 850,344 calls were made to Lifeline during the study period. After excluding calls that were from blocked numbers, of very short or unknown duration, or in certain non-crisis categories (e.g., hang-up and unwelcome calls, donation enquiries and feedback calls), we were left with 411,725 calls in our analysis dataset. These calls were made by 98,174 individuals. Individuals who met the definition of being a frequent caller made up 3 % of all callers (2594) but made 60 % of all calls (247,547) (see Fig. 1). We identified a number of predictors of being a frequent caller, including being male or transgender, and never having been married. The odds of being a frequent caller increased with age until 55–64 years, and then declined. Suicidality, self-harm, mental health problems and issues related to crime, child protection and domestic violence were all associated with being a frequent caller.

#### Data from a purpose-designed survey/interview study with frequent callers to Lifeline

Recruitment occurred during 54 shifts at the two Lifeline centres. Approximately 900 callers were screened for eligibility, and 317 took part in the survey. Of these 317 participants, 69 (22 %) self-identified as frequent callers. They were relatively evenly split in terms of gender (45 % male, 55 % female) and nearly two-thirds (61 %) were aged 45–65 years. When they were asked about what prompted their call to Lifeline on this occasion, the most common responses were: “I regularly call Lifeline to let them know how I am feeling” (86 %); “I have been feeling very nervous, anxious or depressed” (68 %); “There was nobody else that I could talk to” (59 %); and “I was in an immediate crisis” (39 %).

Of the 69 frequent callers who provided survey responses, 19 participated in an interview. Twelve participants (63 %) were female and 11 (58 %) were aged 45–65 years. Fifteen (79 %) reported being unable to work, and 13 (68 %) reported living alone. The interviews suggested that three distinct types of frequent callers exist: addicted callers (who call out of habit and are unable to resist the urge to call); support-seeking callers (who call looking for emotional support as they are unable to cope with constant life stressors); and reactive callers (who call when they become unsettled by an external trigger). In addition, the interviews pointed to some themes that were common to all callers. These drove their frequent use of the service and included: positive reinforcement; social isolation; anonymity; and unrestricted access.

#### Data from the *diamond* study

Data on telephone helpline use was available for 713 (90 %) of *diamond* participants in the first year. In total, 16 participants (2 %) reported frequent use of telephone helplines. Frequent use of telephone helplines was associated with being relatively young (18–34 years), having difficulties managing on available income, and several indicators of social isolation (e.g., living alone and being bothered a lot by not having a confidant). Several physical health factors were also predictive of frequent use (e.g., having a chronic disease and/or self-rating of own health as poor or fair), as were a number of mental health factors (e.g., having anxiety, major depression, a likely personality disorder and/or suicidal thoughts, and/or using antipsychotic medication). Frequent use of telephone helplines was also associated with using emergency departments, psychologists and psychiatrists. It was also associated with an increased likelihood of visits to more than one general practitioner (GP). In addition, frequent use was associated with greater levels of dissatisfaction with access to health services.

#### Data from Australia’s NSMHWB

In total, 90 respondents reported using telephone counselling services in the past 12 months. Forty-seven per cent did so once (one-off callers) and 53 % did so on repeat occasions (24 % twice, 17 % 3–6 times, and 12 % ≥7 times). We compared repeat callers with one-off callers and found that the former were more likely to have an anxiety disorder, to consult GPs and allied health professionals for mental health problems, and to be unemployed.

## Discussion

### Summary of findings

Our empirical studies add significantly to current knowledge about frequent callers to telephone helplines. Most of them are larger in scale and more rigorous than their predecessors. They explicitly consider differences between frequent callers and one-off or episodic callers. Collectively, they allow us to triangulate our findings and consider whether there are some findings that appear to be consistent across studies with different methodologies and data sources. They provide a similar picture of frequent callers, and thus give us confidence that the profile that we have identified is valid.

Several key findings stand out. Frequent callers are relatively few in number but they account for a substantial proportion of calls. They have a heavy reliance on helplines, perhaps because they are isolated and have relatively few social supports. They are by no means just “time wasters”, however; they have high levels of need, as evidenced by the fact that they have major mental health problems (including anxiety, depression and suicidality) and are often in crisis. They also make use of other services for their mental health problems, including GPs, allied health professionals (e.g., psychologists), psychiatrists and emergency departments. The circumstances under which frequent callers make use of telephone helplines vary, but current service models are not meeting their needs and are reinforcing their calling behaviour.

### Strengths and limitations

The key strength of this body of work is that it brought together four separate empirical studies to address identified gaps in the existing literature. These studies utilised a range of data sources and employed a variety of different methods, allowing us to triangulate our findings and draw conclusions with a degree of certainty that was not previously possible.

Individually, of course, each of our studies had limitations and these have been acknowledged elsewhere [19–22]. Mostly, these related to the fact that, with the exception of our survey/interview study, we were reliant on routinely collected data (as with our analyses of Lifeline calls data) or data collected in the context of other studies whose original emphasis was not on frequent callers (as with our analyses of data from the *diamond* study and the NSMHWB). This sometimes meant that particular variables were collected in a way that was not always ideal for our purposes and/or that the numbers of frequent callers were small. This in turn meant that we were not always able to operationalise frequent callers as those who called 20 times or more within a month and/or had to aggregate callers into imperfect groups. With the *diamond* study, for example, we had to deem those who called once a week or more as frequent callers because of the way the data were collected. With the NSMHWB, we were restricted to looking at those who called more than once in a 12 month period (termed repeat callers) as a group, because the numbers of individuals who called at higher rate than this were too small for meaningful analysis.

### Using the findings to inform a potential model of service delivery for frequent callers

The findings from our empirical studies show that the current model of service delivery is not working for frequent callers. Features of the model—e.g., the fact that callers remain anonymous and can make unlimited calls—reinforce their calling patterns. An alternative model is required that better serves the needs of both frequent callers and other callers who use telephone helplines episodically or in a one-off manner. We propose a new model here.

The proposed model is guided by the following principles:

- The model addresses a problem that is about behaviours (frequent calling) not individuals (frequent callers). The existence of these behaviours provides evidence that these callers’ needs are not currently being met. These behaviours present a challenge for telephone helplines but it may be possible to “turn them around”. Those engaging in these behaviours may benefit from a different approach.
- The model is non-judgemental. It recognises that frequent callers have high levels of genuine need. They have complex mental and physical health problems and a range of social issues, and experience crises that may not be quickly resolved but instead may be more ongoing in nature and may be heightened by specific triggers or at times of stress and anxiety. They are isolated, but that this is not the sole reason for their calling patterns.
- The model recognises that individuals are different and therefore offers flexibility and choice. Some frequent callers will “opt in” and take advantage of the new model, whereas others may prefer not to.
- The model articulates clear roles and responsibilities for frequent callers who do choose to use the new model of service delivery. It empowers frequent callers by involving them in early decisions about their ongoing care (e.g., goals) and commits them to calling at agreed times.
- The model acknowledges that frequent callers are accessing a range of other services in addition to telephone helplines. It capitalises on this, and promotes collaboration between telephone helplines and these other services wherever possible and appropriate.

The features of the model, and some of the conditions that would necessarily have to underpin them, are described below.

#### Dedicated and specially trained TCSs

Under the proposed model, a sub-group of TCSs would be dedicated to taking calls from frequent callers. They would receive additional training for this, over and above the normal training offered to TCSs. They would also have ongoing opportunities for continued professional development, one-to-one supervision sessions, debriefing, and peer support.

The training and other support offered to these TCSs would focus on equipping them to deal with mental health issues (particularly anxiety, depression and suicidality) and social issues (particularly loneliness and isolation), and on the overlap between these and physical health problems. It would also prepare these TCSs to deal with some of the complexities underpinning frequent callers’ behaviour, including, for example, attachment issues.

The result would be that the telephone helpline would have a cadre of highly skilled TCSs with specific expertise in dealing with frequent callers. Their role would be akin to that of a mental health counsellor in other community settings. These TCSs would cover a significant number of shifts in any given week, and be available at times that frequent callers are particularly likely to call (e.g., at night).

Consideration might be given to whether these TCSs should be retained on a paid basis, rather than as volunteers, and whether individuals with some tertiary or equivalent training in dealing with mental health issues might be preferred over lay people.

#### An integrated, tailored service

Frequent callers would be allocated to one of these specialised TCSs who would develop a rapport with them, establish rules about the timing and duration of their calls, and help them work towards clearly defined goals. The caller and the TCS would reach an agreement about how often the caller could use the service, the type of care he or she should expect to receive, and what to do in the case of an emergency. The TCSs would provide a more intensive, high level of counselling than the standard telephone helpline service.

The TCSs would work with these callers to agree on well-articulated management plans, based on their history and presenting issues. Then, in accordance with these plans, they would assist them to develop strategies for dealing with their various mental health and social issues. They would also explore callers’ relationships with the telephone helpline and guide them towards more adaptive relationships by modelling secure attachment behaviour [37], setting defined boundaries, and articulating clear consequences of breaching these boundaries. They could potentially draw on a range of therapeutic approaches. For example, cognitive behavioural therapy (CBT) [38] could be used to help individuals identify and address maladaptive cognitions and behaviours that motivate their calling behaviour. Alternatively, acceptance and commitment therapy (ACT) [39] could be used to assist callers to come to terms with unpleasant thoughts that drive them to call telephone helplines, and develop strategies to reduce their likelihood of acting on these thoughts.

Calls would take the form of a series of sessions at which particular issues might be discussed or particular strategies might be taught. At each call, the TCS would ask the caller how things are going with issues that were discussed on the previous call, and would follow up with any homework tasks that might have been set. The TCS would acknowledge previous conversations but would guide the caller away from ruminative thought processes.

The model would also draw on newer technologies that might facilitate greater levels of care without being too resource intensive (e.g., the issue of loneliness might be addressed through a facilitated webinar group that meets to discuss topics related to wellbeing, and symptoms of anxiety and depression might be addressed via mobile applications or interactive websites, of which there are many examples [40]). Of course, approaches involving newer technologies might not suit all callers.

#### Linkages to other services

The model might be thought of as part of a stepped care process. Some callers might only use this service, whereas others might be assisted to “step up” to other services, beginning with primary care and, if necessary, moving up to specialised mental health services. This would require good linkages between the telephone helpline and other service pathway elements.

The model recognises that frequent callers are likely to already be using a range of these other services, including GPs and mental health specialists. It is not about creating new linkages but improving the quality of existing ones, reducing reliance on multiple providers (e.g., several GPs), and fostering consistent approaches. For example, there would be instances in which the TCS might work with the caller and his/her GP on a shared care plan.

#### A seamless triage system

For the model to work, a seamless triage system would need to be put in place. Frequent callers would be identified by a variety of means (e.g., through the helpline’s telephony system “flagging” their telephone number, or through cues that they give when they introduce themselves). Once identified, they would be offered the opportunity to speak to one of the dedicated and specially trained TCSs. If they took this up, they would be put through to the TCS who would explain the service to them in more detail and invite them to make use of it. For those who chose to “enrol” in the service, this would act as the first session.

#### Rules of engagement

The ongoing relationship between frequent callers and specific TCSs would require that callers relinquish their anonymity and give their names and contact details. The TCSs would also be required to give their names, although they might use pseudonyms. This open use of names would foster rapport and trust, and would make for more “normal” conversations. It would also be necessary for practical reasons, in order to ensure that the caller could always make contact with his or her allocated TCS.

As noted above, frequent callers making use of the integrated, tailored service would enter into an agreement with the TCS about the timing of their calls. Effectively, they would be calling at agreed appointment times, some of which would be made available after business hours. Callers in an acute crisis could call outside these times, but would be diverted back to the regular service. This arrangement would need to be explained to callers in a manner that did not encourage further frequent use of the helpline, but empowered them to move towards recovery. Further work is required to determine the optimal approach to restricting frequent callers’ calls in this way, and it is likely that the solution will be different for different callers. One option, for example, might be to taper the calls over time, initially allowing callers to call daily, then 2–3 times per week, then weekly, then fortnightly, then monthly etc.

Although the caller would be paired with an individual TCS, the fact that there would be a critical mass of these specialised workers would mean that callers could potentially shift from one TCS to another if they felt that would be beneficial. Equally, TCSs might cross-refer callers, because it is likely that individual TCSs might develop expertise for dealing with particular types of frequent callers, and effectively become super-specialists.

#### Getting the balance right

The proposed model would need to be implemented in a way that ensures that it does not amplify frequent callers’ reliance on telephone helplines. It should: be viewed as an intensive but time-limited service that helps frequent callers to move on with their lives; provide an opportunity for callers to develop a meaningful connection with a specific TCS without encouraging further dependency; and be seen as an alternative to their regular use of the telephone helpline, rather than as an adjunct to it (although, as noted above, it will be necessary to allow callers to make a standard call to the helpline in an emergency). The nomenclature around the model would also require careful thought; it would need to be non-stigmatising without “rewarding” frequent callers by offering them a specialised service.

### Suggested next steps

We would suggest that the next step in the process of dealing with frequent callers is to further refine the proposed model, testing the concept out with key stakeholders. There may be elements that are missing, or existing elements that are seen as unworkable. These stakeholders should include frequent callers themselves, TCSs, supervisors and managers, and representatives from primary care and specialist mental health services. Frequent callers would clearly have a view as to whether this sort of model would address their needs, and our survey/interview study suggests that they are very willing to participate in relevant information-gathering exercises. TCSs would provide valuable insights into the extent to which the model might alleviate some of the stresses associated with dealing with frequent callers, as well as views about whether the model might introduce new issues (e.g., by creating a parallel system of service delivery). Supervisors and managers would provide input from organisational/systems perspectives, and would be able to comment on the workability of the proposed model. Primary care providers (particularly GPs) and specialist mental health providers would have views on how best to formalise collaborative relationships.

One approach to involving stakeholders in the model’s refinement might be to use a co-design methodology [41]. This has been used in other areas of health and social care to reconfigure service systems to better address needs. It emphasises the experiences of stakeholders—particularly users—with the current system. It recognises that service users are not passive recipients of services but instead are integral to ideal models of care.

Once input from frequent callers and other stakeholders has been received and the model has been further refined, it should be tested in a controlled way. This is crucial because the model is not without risks; although it is intended to reduce the reliance of frequent callers on telephone helplines and free up resources for other callers, it is possible that it could have negative impacts. We would recommend piloting the model in a few telephone helpline centres in the first instance, and evaluating it in a methodologically rigorous way. Ideally, this would involve a randomised controlled trial in which frequent callers were randomly allocated to receive the tailored, integrated service or to receive usual care. The trial would consider both the effectiveness of the new service (i.e., its achievement of benefits for frequent callers) and its cost-effectiveness (i.e., weighing these benefits up against the cost of implementing the new service). Careful examination of unintended consequences would also be important. For example, it would be necessary to monitor the total number of calls made by frequent callers allocated to the new service to ensure that they were not in fact making greater use of the given telephone helpline (i.e., using the new service and the regular service in tandem). In addition to focusing on outcomes, the trial should also monitor the processes associated with the new service (e.g., the way in which each of its elements is operationalised) and impacts (e.g., the effects the service has on the telephone helpline as a whole and its TCSs in particular). It should also have an emphasis on the amount and level of training required by the dedicated TCSs.

Lifeline has expressed interest in exploring the potential of the model further, following the sorts of steps we have described above. If the model proves to be successful in this context, consideration might then be given to how to optimise it and ensure that it is replicable and scalable in other services, either in Australia or overseas. Implementation science literature suggests that consideration of the different operational frameworks and different skill sets and experiences of TCSs will be important here [42].

## Conclusions

Around the world, telephone helplines are struggling to deal with their frequent callers. Lifeline has expressed interest in further refining the model we have proposed here. If the model proves to be a successful solution to responding to frequent callers, this will be ground-breaking. The model is based on the best available evidence regarding who frequent callers are and what motivates their calling patterns. We are confident that it (or a modified version of it) could provide the answer to how to meet the needs of frequent callers, as well as those of other callers, TCSs and telephone helpline managers.

## Abbreviations

TCS: telephone crisis supporter
*diamond*: Diagnosis, Management And Outcomes Of Depression In Primary Care
NSMHWB: National Survey of Mental Health and Wellbeing
CBT: cognitive behavioural therapy
ACT: acceptance and commitment therapy
